# Review of Phytochemicals Present in Psidium guajava Plant and Its Mechanism of Action on Medicinal Activities

**DOI:** 10.7759/cureus.46364

**Published:** 2023-10-02

**Authors:** Dhanshree M Joshi, Swanand S Pathak, Shraddha Banmare, Sweza S Bhaisare

**Affiliations:** 1 Clinical Research, School of Allied Health Sciences, Jawaharlal Nehru Medical College, Datta Meghe Institute of Higher Education and Research, Wardha, IND; 2 Pharmacology, School of Allied Health Sciences, Jawaharlal Nehru Medical College, Datta Meghe Institute of Higher Education and Research, Wardha, IND

**Keywords:** guava, psidium guajava l. (myrtaceae), medicinal activities, phytochemicals, herbal remedies

## Abstract

For centuries, herbal remedies have been employed to address a variety of human ailments, and *Psidium guajava* Linn (Myrtaceae), commonly known as guava, stands out as a noteworthy medicinal plant with significant pharmacological potential. In India, particularly in rural areas where access to conventional medicines can be limited, the various parts of the *Psidium guajava* plant, including its leaves, bark, roots, and fruit, have been harnessed for their therapeutic properties to tackle various health issues. *Psidium guajava* Linn proves to be a valuable repository of essential nutrients along with bioactive compounds such as α-terpineol, β-caryophyllene (trans-caryophyllene), rutin, α-humulene, oleanolic acid, flavonoids, and quercetin. These components exhibit diverse medicinal activities, encompassing anti-inflammatory, anti-cancer, anti-bronchitis, anti-proliferative, anti-tumor, anti-bacterial, and anti-diabetic effects. Every facet of the guava plant holds economic significance and is cultivated on a large scale. Taxonomically, *Psidium guajava* can be classified within the Plantae kingdom, Magnoliophyta division, Magnoliopsida class, Rosidae subclass, Myrtales order, Myrtaceae family, Myrtoideae subfamily, Myrteae tribe, Psidium genus, Guajava species. This adaptability of guava to various soils and environmental conditions facilitates relatively easy cultivation, yielding rapid fruit production. Its widespread cultivation across India is attributed to its manifold commercial applications. To comprehensively comprehend how this plant can effectively address the array of health challenges encountered by the Indian populace, this review delves into its multifaceted therapeutic properties, highlighting its significance in healthcare practices. Ongoing research endeavors by investigators continue to uncover novel medicinal attributes associated with *Psidium guajava*, enriching our understanding of its potential benefits.

## Introduction and background

Reaching a height of up to 35 feet, the guava tree thrives in tropical regions and is renowned for its abundant fruit production. Cultivated predominantly in tropical climates, this tree belongs to a diverse botanical family, encompassing over 3,800 species spread across more than 133 genera [1]. The utilization of medicinal plants has significantly contributed to the progress of human health and overall well-being [2]. Residing within the Myrtaceae family, *Psidium guajava* Linn is primarily distributed across equatorial and subtropical regions [3]. The leaves as a part of the plant are used to treat a variety of illnesses, which are followed by the supplementary fragments of the plant being fruit, bark, and finally the tuber at the end [3].

The guava tree prospers in a realm of soils, procreates easily, and produces fruits swiftly, which has led to its widespread expansion throughout the tropics [4]. Rumors circulate regarding the integration of specific components of *Psidium guajava* into traditional medicine practices. Various elements of the plant, including bark, roots, fruits, and leaves, have been identified as possessing diverse pharmacological attributes that can effectively address a multitude of conditions [5].

Tropical medicinal plant species are renowned for their abundance of essential oils containing valuable therapeutic properties and biologically active secondary compounds. The primary advantages attributed to the therapeutic application of botanicals in treating diverse illnesses encompass their affordability, effectiveness, safety, and ready availability. Traditional healers consistently incorporate medicinal plants into their daily practice, drawn by these advantageous attributes. In alignment with the World Health Organization (WHO), approximately 80% of individuals in economically challenged nations depend on indigenous medicine to meet their fundamental medical necessities. This reality holds for the Democratic Republic of the Congo (DRC), where plants play a pivotal role in catering to the healthcare needs of both urban and rural populations, owing to the often prohibitive costs associated with conventional pharmaceuticals [6].

## Review

Methodology

In delving into the multifaceted realm of *P. guajava*, commonly known as guava, and its profound array of medicinal benefits, a systematic and comprehensive review methodology is crucial. This methodology aims to extract and evaluate pertinent information from diverse sources to provide a comprehensive understanding of the subject matter. The review will commence with an extensive literature search across scientific databases, academic journals, and reputable online resources, employing a wide range of keywords such as "*P. guajava*," "guava medicinal properties," and "health benefits of guava." The retrieved studies will be meticulously screened based on predefined inclusion and exclusion criteria, focusing on experimental studies, clinical trials, and scholarly articles that present empirical evidence on guava's medicinal attributes. The selected studies will undergo rigorous quality assessment using appropriate appraisal tools to ensure the reliability and validity of the information. Data extraction will involve categorizing and synthesizing relevant findings, identifying patterns, mechanisms of action, and potential applications of guava's medicinal properties. A thematic analysis approach will be employed to extract key themes and insights from the compiled data. This review will not only showcase the diverse health benefits of *P. guajava* but also contribute to the existing body of knowledge by presenting a comprehensive and up-to-date synthesis of its remarkable medicinal potential.

Morphological characters

*Psidium guajava* (Linn.) is an evergreen shrub or tree that grows 6 to 25 feet tall. It has square, downy twigs and wide-spreading branches. Contrasting leaves are produced by twisted branches. The flowers have four to six fragrant, incurved, white petals with golden anthers that are in the leaf axils. When ripe, the fruit is tiny, pear-shaped, three to six cm long, and reddish-yellow [1]. The exotic fruit known as the guava is round, ovoid, or pear-shaped, with an average diameter of four to ten cm and a weight of 100 to 400 g [6]. The below table contains the food values of guava fruit (Table 1).

**Table 1:** Food value of guava fruit.

S. no	Components	Amount of units present
1.	Calories	77-86 g
2.	Moisture	2.8-5.5 g
3.	Crude fiber	0.9-1 g
4.	Protein	0.1-0.5
5.	Fat	0.43-0.7
6.	Carbohydrate	9.1-17 mg
7.	Calcium	17.8-30 mg
8.	Phosphorous	0.30-0.70 mg
9.	Iron	200-400 I.U.
10.	Vitamin A	0.046 mg
11.	Thiamin	0.03-0.04 mg
12.	Vitamin B3	35 I.U.
13.	Vitamin G4	36-50 mg
14.	Riboflavin	0.6-1.068 mg

Phytochemicals

Guava (*Psidium guajava*) is a tropical fruit tree that has been traditionally used for its potential health benefits, and the compounds you mentioned contribute to its curative effects. Here's a breakdown of some of the major bioactive compounds found in guava and their potential health benefits [7]. α-Terpineol: This is a terpenoid alcohol with reported antioxidant and anti-inflammatory properties. It may have potential benefits for respiratory health and relaxation [8]. β-Caryophyllene (trans-caryophyllene): This is a sesquiterpene that acts as a cannabinoid receptor modulator. It has anti-inflammatory and analgesic properties and may have potential applications in pain management and anti-anxiety treatments [9]. Rutin: Rutin is a flavonoid with antioxidant properties. It is known for its potential to support blood vessel health and reduce the risk of cardiovascular diseases [10]. α-Humulene: Another sesquiterpene, α-humulene, has been studied for its anti-inflammatory and analgesic effects. It may also have potential applications in pain management and as an anti-cancer agent [11]. Oleanolic acid (OA): Oleanolic acid is a triterpene with antioxidant and anti-inflammatory properties. It may play a role in protecting the liver and may have potential benefits for skin health [12]. Quercetin: Quercetin is a flavonoid known for its antioxidant and anti-inflammatory effects. It may support cardiovascular health and have anti-allergic properties [13]. Flavonoids: These are a class of polyphenolic compounds found in guava, known for their antioxidant properties. They may help reduce oxidative stress and inflammation in the body [14]. Tannins: Tannins are polyphenolic compounds that can have antimicrobial and antioxidant properties. They may contribute to the astringency of guava fruit [4]. Lectins: Lectins are proteins that can bind to carbohydrates. They may play a role in the immune system and have potential anti-cancer effects [4]. Ellagic acid: Ellagic acid is a polyphenol with antioxidant properties. It may have potential anti-cancer effects and support skin health [4]. Amritoside: This compound is less well-known but is likely to have potential health benefits, considering its presence in guava [4]. Beta-sitosterol: Beta-sitosterol is a plant sterol that may help lower cholesterol levels and support prostate health [4]. Uvaol, oleanolic, and ursolic acids: These are triterpenes with various potential health benefits, including anti-inflammatory and antioxidant properties [12]. It's important to note that while these compounds have been studied for their potential health benefits, consuming guava as part of a balanced diet can contribute to overall health and well-being. As with any natural remedy, it's essential to consult with a healthcare professional for specific health concerns or treatments [7].

Mechanism of action

Anti-cancer Characteristics

The putative anticancer drug α-T inhibits NF-kB signaling. The transcription factor activates a protein complex called nuclear factor kappa-light-chain enhancer of activated B cells (NF-kB) and controls transcription. Chronic inflammation and cancer development are related (by way of DNA). Blocking NF-kB proved successful in reducing the expression of several different types of human malignancies, which is recommended to make it more sensitive to the effects of anti-tumor medications or to inhibit the growth brought on by cancer cells [15].

Anti-bronchitis Properties

The tumor necrosis factor (TNF) receptor-associated factor 2 interacts with NIK (NF-KB-inducing kinase) and MAP (a member of the mitogen-activated protein) kinase family, causing NF-kB activation and IkB degradation by multi-receptor TNF. A real family member protein heterodimer, NF-kB consists of p65 (RelA), which is deactivated by its association, and p50 (NFKB1) using inhibitors of the IKB family. IKB is chosen by the body to be a ubiquitinated and degraded proteasome, allowing NF-kB to go into the crux, where it can interact with certain deoxyribonucleic acid (DNA) regions in the target gene promoters. The inhibition of IKK2 of IkB-kinase beta, a gene associated with chronic obstructive pulmonary illness occurrence of disease (COPD) [16].

Anti-inflammatory Activity

The prime inflammatory medium, inducible nitric oxide synthase (iNOS), is inhibited by β-caryophyllene (BCP), which seeks to have anti-inflammatory effects. Nitric oxide itself activates soluble guanylyl cycles, which results in the creation of current good manufacturing practices (cGMPs) (good manufacturing practices). The soluble guanylyl cyclase activates is a frequent route in numerous processes, including vascular smooth relaxation of muscle cells, suppression of platelet and neutrophil activity chemotaxis, as well as central and peripheral nervous system signal transduction [17]. The equilibrium between iNOS induction and suppression may be the source of a lot of the inflammation's pathology and physiology [18]. Reactive species of nitrogen oxide (RNOS), NO, and reactive oxygen species (ROS) are substances that directly contribute to the activation or suppression of important metabolic process enzymes, including mitochondrial breathing and DNA synthesis [19].

Anti-diabetic Behavior

Rutin increased the absorption of glucose and added insulin for glycemic control. The activation of the insulin signaling pathway-promoting receptor kinase led to enhanced glucose absorption and elevated GLUT4 translocation [20]. The glucagon, a heterotetrameric glycoprotein called receptor, is made up of the following subunits: both α and β components. It indicates that the α-subunit is completely extracellular and consists of an insulin-binding site. As a result, the transmembrane proteins in the β-subunits are the intracellular signaling components [21]. The insulin receptors and monetary units are descended from a single-chain precursor or proreceptor, etc. [22]. Tyrosine is stimulated by insulin after it binds to the α-subunit. Phosphorylation of the insulin receptor's β-subunit. In this scenario, adenosine triphosphate (ATP) serves as the phosphate donor, and only on tyrosine residues does phosphorylation take place, namely histone 2B several cytoskeletal proteins, glycolytic enzymes, and tyrosine-containing peptides are also present. Instead of altering the catalytic velocity (V.m), insulin activates the kinase via substrate compatibility. Thus, the elimination of the subunit's insulin binding domain via either a light tryptic treatment or in vitro mutagenesis results in the kinase being activated to mimic the action of the insulin [23].

Anti-tumor Activity

Oleanolic acid's anti-tumor or anti-cancer properties in the case of the development of tumor and cancer models (in vivo and in vitro). Oleanolic acid (OA) prevents the development of the transplanted tumor and the expansion of hepatocellular cells in the liver (HepG2). The tumor upregulation is caused by OA's antitumor action. Cyclooxygenase-2 (COX) and protein (p53)-mediated activation of mitochondrial cell cycle arrest and the apoptotic pathway [24]. The second mitochondrial-derived activator of caspases (SMAC) mimics BV6 and OA administration results in the activation of cell death in mortal hepatocarcinoma [25]. SMAC is a synthetic selective antagonist of apoptosis inhibitors called mimetic BV6 (IAP) proteins and is a potential analeptic contender for the treatment of cancer. The induction of apoptosis has been proposed as the OA's anti-tumor property mechanism. Regarding miR-122 overexpression, a protein that has been identified as crucial as a tumor suppressor in some cancer types [26], OA exhibits anti-cancer properties in human cervical malignancy, bladder tumors, lung cancer, and breast melanoma cells. In general, OA demonstrates therapeutic compounds against tumors, such as anti-tumor and anti-cancer, through their various mechanisms of action [25].

*Anti*-*bacterial Activity*

Quercetin's antibacterial mechanism primarily involves breaking down the bacterial cell wall, altering the permeability of the cell, interfering with protein compounds, and expression, lowering enzyme activity, and preventing the synthesis of nucleic acids. Quercetin was able to be sulfated and phosphorylated at various hydroxyl groups and adjusted its antibacterial capabilities to increase or decrease its solubility, and as a result, for particular bacterial species [27], the ability of quercetin to fight germ derivatives against various bacterial species took place at various minimal antagonistic concentrations (MICs). Quercetin demonstrated its antimicrobial power using antibiotics in combination [28].

Anti-proliferative Activity

They specifically target kinases that phosphorylate specific regions on proteins. Phosphoinositide 3-kinase, Akt/protein kinase B (PKB), and other signaling pathways are all affected by flavonoids. Protein kinase B (PKB), protein-1 kinase C (P1KC), and mitogen-activated protein (MAP) kinase are examples of these enzymes. Flavonoids' stimulatory and inhibiting effects on these pathways change the target's phosphorylation to modify how the cell operates molecules and by altering the way genes are expressed. Flavonoids could impact growth. Signaling by preventing growth factor or receptor phosphorylation rheostat binding additionally, flavonoids block Lck (lymphocyte-specific protein kinases), Fyn (proto-oncogene tyrosine-protein kinase), and protein tyrosine kinase are two representative nonreceptor kinases from the steroid receptor coactivator (SRC) family, engaged in trafficking of T cells signaling. The metabolites of flavonoids continue to have the capacity to interact with the signaling pathway's proteins. The involvement of flavonoids in cell signaling might be one of the factors responsible for their anti-proliferative activities [29].

Traditional uses of each part of the plants' *Psidium guajava*

The below table consists of the uses of each part of the plant *Psidium guajava* (Table 2).

**Table 2:** Traditional uses of each part of the plant Psidium guajava.

Parts of the plant	Uses
Leaves	Used as an anti-inflammatory agent. Effective against spasms. Beneficial for rheumatism. Can be used to treat bronchitis, asthma attacks, cough, and lung diseases [29].
Bark	Formulation from the bark is used as an astringent. Applied to heal bed sores. It helps in cases of diarrhea and dysentery. Used to treat skin diseases. Applied for skin sepsis. May be beneficial in treating menorrhagia (heavy menstrual bleeding), fevers, and pulmonary diseases [7].
Roots	Relieving reflux. Treating cough. Alleviating abdominal pain. Managing dyspepsia (indigestion). Addressing constipation. Relieving toothache [7].
Fruit	Medication for cholera. Treating diarrhea and dysentery. Aiding in indigestion. Providing relief from constipation. Beneficial for pharyngitis (inflammation of the throat) and laryngitis. Used in managing skin disorders and ulcers [7].
Aerial parts	The entire guava plant or its branches are utilized as a skin tonic in the form of pulp. Useful in treating bruises. May have applications in managing menstrual disorders. Traditionally used for cases of miscarriages and preterm labor pains [30].

## Conclusions

*Psidium guajava* stands as a botanical marvel, offering a diverse array of medicinal benefits that span from immune enhancement to potential cancer prevention. Its versatility as a natural remedy, coupled with its delightful taste, makes it a unique and compelling choice for individuals seeking to optimize their health through holistic means. The therapeutic and prophylactic use of allopathic medications for illness has resulted in the quick emergence of drug resistance all over the world. Resistance to natural therapy or ayurvedic therapy, on the other hand, is extremely unusual, which has prompted many people to move from allopathic to ayurvedic medicine. However, extracting the active element from the crude natural substance poses a significant barrier for researchers, necessitating the development of a streamlined approach. Natural therapy is not only safe and easy to obtain, but it is also cost-effective in the treatment of disease. The fruit of the *Psidium guajava* plant, along with its juice, is widely used because of its nutritional benefits. As scientific exploration continues, the significance of *Psidium guajava* in the realm of medicine is bound to flourish, reaffirming its position as a treasured gift from nature.
